# Benzoic acid versus salicylic acid efficacy in regulating growth, accumulation of secondary metabolites and NO_3_ content of Swiss chard (*Beta vulgaris* L. var. *cicla*)

**DOI:** 10.1186/s12870-026-09593-5

**Published:** 2026-07-28

**Authors:** Nora A. AbdelMotlb, Gamal S. Riad, Dina M. Salama, Sabry M. Youssef, Salama A. Abd Elhady, Mostafa G. Shahin, Hani S. Saudy, Amr A. Metwally

**Affiliations:** 1 Vegetable Research Department, Agricultural and Biology Research Institute, National Research Centre, El Buhouth St, Cairo, 12622 Egypt; 2 Department of Horticulture, Faculty of Agriculture, Ain Shams University, Cairo, 11566 Egypt; 3 Agronomy Department, Faculty of Agriculture, Ain Shams University, Cairo, 11566 Egypt

**Keywords:** 2-hydroxybenzoic acid, Benzenecarboxylic acid, Hormonal balance, Nitrate level, Nutritional status, Swiss chard quality

## Abstract

**Purpose:**

Salicylic acid (SA) and benzoic acid (BA) are important phenolic regulators involved in plant growth and defense; however, their comparative roles in modulating growth and secondary metabolites production in leafy vegetables remain unclear. Therefore, this study aimed to investigate the influence of exogenous SA and BA on growth, mineral composition, and bioactive compound accumulation in Swiss chard (*Beta vulgaris* L. var. *cicla*).

**Methods:**

Field experiments were conducted over two successive seasons of 2023/24 and 2024/25 to assess the influence of SA and BA on Swiss chard cv. Ruby Red. Plants were treated with SA and BA at 1, 2, and 3 mM, each alongside an untreated control. Treatments were arranged in a randomized complete block design with three replicates. Beside the agronomic attributes, photosynthetic pigments, total phenols, total flavonoids, total antioxidant activity, total indoles, anthocyanin, nitrate, and mineral content were assayed.

**Results:**

Both SA and BA significantly improved growth parameters, mineral content, and secondary metabolites accumulation compared to the control. SA at 1 mM or BA 1 mM were the most effective treatments, showing the highest significant values of vegetative parameters, photosynthetic pigments, total phenolics, total flavonoids, total antioxidant activity, total indoles, anthocyanin, and mineral content. Further, BA 1 mM was the potent treatment for lowering leaf nitrate content, showing the lowest nitrate content, which was significantly lower than SA at 2 and 3 mM. Correlation analysis revealed strong positive relationships between growth parameters and secondary metabolites.

**Conclusion:**

BA behaves influence similar to SA for enhancing leaves yield with higher efficacy in reducing nitrate content via enhancing the efficiency of photosynthetic pigments and regulating various secondary metabolites. Herein, BA at low concentration (1 mM) is a promising rate to be applied to obtain high yield with good quality.

**Supplementary Information:**

The online version contains supplementary material available at 10.1186/s12870-026-09593-5.

## Introduction

Swiss chard (*Beta vulgaris* L. var. *cicla*), a biennial leafy vegetable within the Amaranthaceae family, which is extensively consumed, owing to its considerable nutritional profile and the presence of numerous bioactive constituents, including phenolic compounds, flavonoids, betalains, carotenoids, and vitamins [1–3]. These phytochemicals are implicated in a range of biological functions, encompassing antioxidant, anti-inflammatory, antidiabetic, and hepatoprotective properties [4, 5]. Phenolic acids contribute strongly to antioxidant capacity in Swiss chard extracts [1]. Phenolic compounds are the largest group of secondary metabolites in plants, which originate from phenylalanine and are therefore referred to as phenylpropanoids [6, 7]. Phenols include many groups, i.e., phenolic acids, flavonoids, stilbenes, and lignans. Phenolic compounds are mainly synthesized biogenetically through the shikimate/phenylpropanoid pathway [8, 9]. Phenolic compounds are present throughout the tissues of higher plants, where they function as signaling molecules in specific symbiotic relationships and serve as defensive agents against soil pests and pathogens [10]. In Swiss chard, significant secondary metabolites encompass phenolic acids, flavonoids, betalain pigments, and carotenoids, all of which contribute to antioxidant activity and nutritional value [2].

Among plant signaling molecules, salicylic acid (SA) and benzoic acid (BA) have gained attention as effective elicitors of secondary metabolism under abiotic and biotic stress conditions [10–12]. Salicylic acid (2-hydroxybenzoic acid) is a phenolic derivative compound that exists naturally in plants [13] and acts as an endogenous regulator, similar to a hormone, that affects a wide range of processes in plants. SA is implicated in several aspects of plant growth, development, and physiological processes; stomatal closure, photosynthesis, pigment accumulation, and nutrient uptake [14]. Furthermore, SA is known to initiate plant defense mechanisms in response to both biotic and abiotic stressors [15–17], which subsequently results in the accumulation of secondary metabolites, encompassing bioactive compounds like phenolics, flavonoids, alkaloids, and terpenoids [18, 19]. In addition, SA improved physiological and quality parameters in Swiss chard grown in aquaponic systems; the total phenolic compounds and antioxidant capacity were increased, indicating enhanced secondary metabolism [20]. In addition, a study of SA on the Swiss chard plants indicated that salicylic acid stimulates biosynthetic pathways responsible for secondary metabolites and antioxidant compounds, hence it increased the chlorophyll, carotenoids and phenolic compounds content [21].

Benzoic acid (BA) is an organic aromatic monocarboxylic acid that is synthesized *via* reactions of the phenylpropanoid pathway. BA is a key biosynthetic precursor to SA, methyl benzoate, benzoylated glucosinolates, and many phenolic derivatives [22]. BA plays an important role in defense, signaling, and environmental interaction [11, 12, 22, 23]. Furthermore, BA derivatives have been reported to enhance secondary metabolite biosynthesis by increasing the metabolic flux through phenylpropanoid pathways [24]. At low concentrations, BA showed promotive effects on growth and productivity through enhancing auxin production and cell division. However, it has inhibitory effects at high concentrations, acting as allelochemical, inhibiting root development and decreasing plant biomass [25].

Although salicylic acid (SA) has been reported to enhance the growth and phytochemical composition of plants, the role of benzoic acid (BA), particularly as a metabolic elicitor, remains largely unexplored. To date, no study has comprehensively compared the effects of SA and BA on the growth, mineral nutrition, nitrate accumulation, and biosynthesis of secondary metabolites in Swiss chard under open-field conditions. This knowledge gap is significant because BA, as the biosynthetic precursor of SA and a structurally related phenolic compound, may induce similar physiological and biochemical responses while exhibiting distinct effects on nitrate accumulation and metabolic regulation. Therefore, the present study aimed to comparatively evaluate the influence of foliar application of SA and BA on plant growth, mineral composition, nitrate content, antioxidant capacity, and the accumulation of bioactive secondary metabolites in Swiss chard, thereby providing new insights into the potential of BA as an alternative biostimulant for improving crop productivity and nutritional quality.

The current research hypothesized that exogenous application of SA and BA differentially modulates plant growth and phenylpropanoid-derived secondary metabolites accumulation. Therefore, this study was designed to comparatively evaluate the effects of foliar application of salicylic acid and benzoic acid on growth, accumulation of secondary metabolites, i.e., phenolics, flavonoids, and anthocyanin, and antioxidant activity, total indoles, and mineral content in Swiss chard (*Beta vulgaris* L. var. cicla cv. Ruby Red, to elucidate their potential roles as metabolic elicitors.

## Materials and methods

### Study site attributes

A field experiment was conducted at the trial farm Hort. Dept., Fac. Agric., Ain Shams Univ., Egypt (Latitude 30°29’56.9"N 30°19’12.9"E, and mean altitude 21 m above sea level), during the 2023/24 and 2024/25 seasons. Soil samples were taken at a depth of 0 to 30 cm and analyzed to determine the physico-chemical profile of the soil [26, 27] as presented in (Table 1). Meteorologically, the study had cool winter with averages of weather data, i.e. maximum temperature, minimum temperature, relative humidity, wind speed and solar radiation of 30.1 °C, 15.8 °C, 47.5%, 2.1 m s^− 1^ and 28.2 MJ m^–2^ day^–1^, respectively.

**Table 1:** Initial physico-chemical properties of the soil at the trial farm before Swiss chard cultivation

Property	Unit	Value
Mechanical analysis		
Sand (%)	%	23.80
Silt (%)	%	35.90
Clay (%)	%	40.30
Soil texture		Clay
Chemical analysis		
pH value		7.25
EC at 25 °C	dS m^− 1^	0.54
Calcium	meq L^− 1^	0.59
Magnesium	meq L^− 1^	1.75
Sodium	meq L^− 1^	0.46
Potassium	meq L^− 1^	1.16
Chloride	meq L^− 1^	0.54
Carbonate	meq L^− 1^	-
Bicarbonate	meq L^− 1^	0.27

### Plant materials

Salicylic acid (SA) and benzoic acid (BA) were purchased from Sigma-Aldrich (Merck, Darmstadt, Germany), with molecular weights 138.12 and 122.12, respectively. Both of SA and BA were dissolved in 100 µL dimethyl sulfoxide (DMSO) as a solvent and the concentrations (1, 2, and 3 mM) of SA and BA were prepared by adding distilled water with a surfactant (Tween 20, 0.02%), and the final concentration of DMSO in the final spray solution was kept constant at 0.01% (v/v) across all treated solutions; the control spray contained distilled water with the same DMSO and Tween 20 concentrations to avoid solvent- or surfactant-related bias. The spraying solution was applied at a rate of 500 L ha^− 1^. Seeds of Swiss chard (*Beta vulgaris* L. var. cicla) cv. Ruby Red were obtained from Holmes Seed Company, USA.

### Trial procedures and design

Foliar sprayings of SA and BA at 1 mM, 2 mM, and 3 mM each, in addition to a control treatment, were performed. The treatments were arranged in a complete randomized block design with three replicates. In the nursery, seeds were sown into cavity-seeding trays and then transplanted after 6 weeks. The open field was prepared, where the trial unit area was 18 m^2^ (6 m length x 3 m width). The seedlings were transplanted in rows, 60 cm distance and 30 cm apart. Accordingly, each experimental unit contained approximately 100 plants. Transplanting was done on 15th October of both seasons and plants were irrigated via a drip irrigation system. The foliar sprayings were applied three times, 20, 55 and 90 DAT. About a month after each spraying, the plants were defoliated to obtain leaves yield.

### Sampling and data recording

#### Agronomic attributes

The central plants were used for data collection to minimize border effects. Plant height, leaves fresh and dry weights, leaves number plant^− 1^, and leaf area were estimated after each instance of plant defoliation, 50, 85 and 120 DAT. Plant height and leaf area were recorded as an average across all defoliations. The leaf disc method is used to calculate the leaf area [28]. A puncture sampler with an area of 3.14 cm^2^ was used to take discs from leaves. Six to ten discs along the leaf blade were collected from each leaf, depending on the leaf size, and 50 discs were collected at each replicate. The whole leaves and the disks were weighed. The leaf area was calculated using formula 1.

The leaves fresh and dry weights, and leaves number were represented as cumulative readings of the three defoliations. To obtain dry weight, the fresh leaves were dried in an oven at 70ºC until a constant weight

1 $${Leaf}\:{area}\:=\:\frac{{Disc}\:{area}\:\times\:\:{disc}\:{numbers}\:\:\times\:\:{leaves}\:{fresh}\:{weight}}{{Disc}\:{fresh}\:{weight}}$$

#### Photosynthetic pigments

At the 3^rd^ defoliation, 120 DAT, five plants were chosen randomly to collect newly fully expanded developed leaves to assess photosynthetic pigments (chlorophyll a, b, total chlorophyll, and carotenoids) colorimetrically. The N, N- Dimethylformamide was used to extract the pigments from the plant samples. The obtained extracts were measured spectrophotometrically at wavelengths of 664, 647, and 470 nm, for assaying chl a, chl b and carotenoids, respectively [29].

#### Secondary metabolites

At 120 DAT, secondary metabolites were assayed Swiss chard leaves. Total phenols were assessed on a dry weight basis, phenolics was extracted by 80% ethanol, phenolics were determined by adding 1 ml of sample, 70 ml distilled water followed by Folin-Ciocalteau reagent, and 15 ml of saturated sodium carbonate solution, incubated at room temperature for 30 min and measured at 765 nm using a spectrophotometer, gallic acid was used to make the calibration curve [30]. Also, total flavonoids was extracted by ethanol 80%, and 0.5 ml of sample, 10% aluminum chloride (0.1 ml), 1 M potassium acetate (0.1 ml), and distilled water (4.3 ml) were mixed. After incubation at room temperature for 30 min, the absorbance was measured at 415 nm using a spectrophotometer Chang et al. [31]. Quercetin was used to make the calibration curve [31]. While, total indoles were estimated adding 1 ml of ethanol extract sample into a test tube followed by 2 ml of salkowski reagent (consisting of 150 ml concentrated H_2_SO_4_ and 7.5 ml FeCl_3_.6H_2_O 0.5 M), This solution was incubated for 30 min at a dark room temperature. Next, total indoles were measured at a wavelength of 530 nm using a spectrophotometer [32].

The total antioxidant activity was determined in the Swiss chard plants, a stock solution was prepared by dissolving 24 mg 1,1-diphenyl-2-picrylhydrazyl (DPPH) with 100 ml methanol and then stored at 20 °C until needed. The solution was obtained by mixing 10 ml stock solution with 45 ml methanol to absorb 1.1 ± 0.02 units at 515 nm. Extracts (750 µL) were allowed to react with 1,500 µL of the DPPH solution for 5 min in the dark. Then, the absorbance was taken at 515 nm. The standard curve was linear between 25 and 800 µmol Trolox [33]. In addition, total anthocyanin content was spectrophotometrically analyzed in a 70% methanol extract. The absorbance of the reaction mixture was read at 520 nm, and the anthocyanin content was expressed as mg cyanidin chloride equivalent per gram of dry weight tissue (mg CYE/g DW) [34]. Moreover, the nitrate content in swiss chard samples was extracted by using hot water extraction, 0.1 g of the dried samples was added to 10 ml of distilled water in test tubes, then it was put in a boiling water path for 20–30 min. Afterwards, 0.25 ml of the extract was placed in a 50 ml conical flask and mixed well with 0.8 ml of 5% (wt/vol) salicylic acid in concentrated sulfuric acid. After 20 min at room temperature, 19 mL of 2 N NaOH (40 g of NaOH pellets were dissolved in 100 mL of water, and transferred to a 500 mL volumetric flask and made up to 500.0 mL with distilled water) were added to bring the level of pH above 12, the samples were cooled at room temperature, and the absorbance was measured at 410 nm [35].

#### Minerals assay

Concerning the mineral assay in Swiss chard, the content of nitrogen, phosphorus, and potassium was estimated in the dry leaf samples obtained at 120 DAT. The dried leaf samples were ground to a fine powder, and digested using sulfuric acid and hydrogen peroxide. Nitrogen percentage was determined using the Kjeldahl method [26]. A spectrophotometer was used to determine the phosphorus content, and it was measured at 800 nm [36]. While, potassium was assayed using a flame photometer [37].

### Statistical analysis

Before combined analysis, assumptions of normality and homogeneity were checked using diagnostic residual plots and Levene’s test. Because the assumptions were acceptable, data from the two seasons were analyzed using a combined randomized complete block model presented in formula 2 [38]. The model included year (Y), replicate nested within year [Rep(Y)], treatment (T), and the Y x T interaction. Year and treatment were considered fixed effects, whereas Rep(Y) was treated as a random blocking effect. Year was tested against Rep(Y), treatment was tested against Y x T, and Y x T was tested against the residual error. CoStat package program (Version-6.303; CoHort Software, USA) was utilized for carrying out the analysis of variance. Mean comparisons were performed using Duncan’s multiple range test at p < = 0.05. Pearson correlation coefficients were calculated and displayed using a heat map, and PCA was performed in R software (Version 4.0.2). For PCA, variables measured in different units were centered and scaled to unit variance before analysis.

2 $$\:{Y}_{ijk}=\mu\:+{Y}_{i}+{R}_{j}\left({Y}_{i}\right)+{T}_{k}+(Y\times\:T{)}_{ik}+{\epsilon\:}_{ijk}$$

Where:

where $\:{Y}_{ijk}$is the observed value of the measured trait in the *\:i*th year, *\:j*th replicate, and *\:k*th treatment; $\:\mu\:$ is the overall mean; $\:{Y}_{i}$ is the fixed effect of the *\:i*th year; $\:{R}_{j}\left({Y}_{i}\right)$ is the random effect of the *\:j*th replicate nested within the *\:i*th year; $\:{T}_{k}$is the fixed effect of the *\:k*th treatment; $\:{\left(Y\times\:T\right)}_{ik}$ is the fixed interaction effect between year and treatment; and $\:{\epsilon\:}_{ijk\:}$ is the residual experimental error, assumed to be normally and independently distributed with homogeneous variance.

This model was applied to test the main effects of year and treatment and their interaction. The year × treatment interaction was used to assess the stability of treatment effects across years. When this interaction was not significant, treatment means were pooled across years. However, when a significant interaction was observed, treatment effects were interpreted separately within each year to identify year-specific responses.

## Results

### Agronomic attributes

Data presented in Table 2 indicate that foliar application of salicylic acid (SA) and benzoic acid (BA) significantly (*p* ≤ 0.05) affected all vegetative growth parameters of Swiss chard plants compared to the control treatment. Year effect was significant for leaves number, leaves fresh weight, and leaf area, but not for plant height and leaves dry weight. Treatment effects were significant for all agronomic traits, whereas the Y x T interaction was not significant for any of these traits. The SA and BA applications at 1 mM were the superior treatments, exhibiting the highest significant values for all vegetative parameters. In this respect, the increases amounted to 13.2% for plant height 34.1% for fresh weight, 40.3% for dry weight, 25.9% for leaves number and 22.1% for leaf area as compared to the control treatment. In addition, BA at 1 mM showed increments of 10.70% in plant height, 30.46% in fresh weight, 38.3% in dry weight, 19.4% in leaves number and 18.7% in leaf area as compared to untreated plants. However, SA at 2 mM significantly improved most parameters, except leaves fresh weight, while BA at 2 mM significantly increased leaves number only. In contrast, SA and BA at 3 mM caused a reduction in growth parameters, approaching control values, particularly in the case of BA at 3 mM.

**Table 2:** Effect of salicylic acid (SA) and benzoic acid (BA) concentrations (1, 2 and 3 mM) on the plant height, leaves fresh and dry weights, leaves number and leaf area of Swiss chard cv. Ruby red plant

Factor	Plant height (cm)	Leaves fresh weight (g)	Leaves dry weight (g)	Leaves number	Leaf area (cm^2^)
Season					
2023/2024	50.75 ± 0.48a	521.43 ± 0.20b	70.74 ± 13.40a	25.44 ± 2.03b	1466.12 ± 28.63b
2024/2025	51.16 ± 0.63a	531.86 ± 0.16a	71.01 ± 12.00a	27.39 ± 1.96a	1496.05 ± 24.38a
Treatment					
SA 1mM	54.06 ± 0.53a	597.67 ± 6.44a	80.65 ± 1.15a	29.16 ± 0.66a	1632.91 ± 17.59a
SA 2mM	52.06 ± 0.53abc	553.83 ± 12.68bc	75.11 ± 0.92ab	27.18 ± 0.33ab	1540.66 ± 35.28ab
SA 3mM	49.89 ± 0.89cde	514.33 ± 17.32d	69.67 ± 3.59bc	24.99 ± 0.51c	1430.78 ± 38.19 cd
BA 1mM	52.89 ± 0.68ab	581.17 ± 9.98ab	79.45 ± 3.35a	27.66 ± 0.66a	1587.83 ± 15.88ab
BA 2mM	51.06 ± 1.12bcd	534.33 ± 3.84 cd	71.08 ± 1.65b	27.51 ± 0.57a	1486.42 ± 18.69bc
BA 3mM	48.94 ± 0.95de	459.33 ± 18.02e	62.71 ± 2.19 cd	25.17 ± 1.44bc	1351.5 ± 33.03d
Control	47.78 ± 0.63e	445.83 ± 8.83e	57.47 ± 1.38d	23.16 ± 0.33c	1337.5 ± 22.5d
*P*-values					
Year (Y)	0.3922ns	0.0416*	0.8404ns	0.0300*	0.0374*
Treatment (T)	0.0045**	0.0001***	0.0007***	0.0116*	0.0005***
Y × T	0.4502ns	0.6428ns	0.5739ns	0.3031ns	0.6511ns

Values are mean ± SE. Treatment means are pooled over the two years and are based on three replicates per year. Year means are based on treatment means across the two seasons. Means within a column followed by the same letter are not significantly different (p < = 0.05) according to Duncan’s multiple range test. ns, *, **, and *** indicate non-significant and significant at p < = 0.05, p < = 0.01, and p < = 0.001, respectively

### Photosynthetic pigments

As shown in Table 3, chlorophyll a, chlorophyll b, total chlorophyll, and carotenoid contents were significantly enhanced by SA and BA treatments compared to the control treatment. Year means and p-values for Y, T, and Y x T. Year effects were significant for chlorophyll a, total chlorophyll, and carotenoids, whereas chlorophyll b did not differ significantly between years. Treatment effects were significant for all photosynthetic pigments, while Y x T interactions were non-significant. SA at 1 mM recorded the highest significant values for chlorophyll a, chlorophyll b, total chlorophyll, and carotenoids which increased by 13.19%, 60.61%, 22.73%, and 31.40%, respectively, compared to the control. Similarly, BA at 1 mM significantly enhanced the photosynthetic pigments and showed same significant effects as the superior application of SA 1 mM. However, similar to the potent two treatments (SA or BA at 1 mM), enhancements in all tested photosynthetic pigments, except carotenoids (with SA at 2 mM) and chlorophyll b (with BA at 2 mM) were obtained. While raising the concentrations of SA and BA at 3 mM resulted in a decline in pigments content.

**Table 3:** Effect of salicylic acid (SA) and benzoic acid (BA) concentrations (1, 2 and 3 mM) on the photosynthetic pigments (mg g− 1 fresh weight) of Swiss chard cv. Ruby red

Factor	Chlorophyll a	Chlorophyll b	Total chlorophyll	Carotenoids
Season				
2023/2024	1.52 ± 0.02b	0.43 ± 0.02a	1.95 ± 0.03b	0.280 ± 0.009b
2024/2025	1.55 ± 0.02a	0.45 ± 0.02a	2.00 ± 0.04a	0.300 ± 0.006a
Treatment				
SA 1mM	1.63 ± 0.01a	0.53 ± 0.03a	2.16 ± 0.03a	0.339 ± 0.008a
SA 2mM	1.59 ± 0.02ab	0.49 ± 0.02a	2.08 ± 0.03ab	0.303 ± 0.011bc
SA 3mM	1.49 ± 0.02 cd	0.48 ± 0.02ab	1.97 ± 0.05b	0.256 ± 0.007d
BA 1mM	1.62 ± 0.01a	0.46 ± 0.02ab	2.09 ± 0.01ab	0.328 ± 0.005ab
BA 2mM	1.53 ± 0.03bc	0.43 ± 0.01abc	1.98 ± 0.02b	0.298 ± 0.013c
BA 3mM	1.45 ± 0.01d	0.37 ± 0.03bc	1.82 ± 0.02c	0.264 ± 0.005d
Control	1.44 ± 0.01d	0.33 ± 0.07c	1.76 ± 0.07c	0.258 ± 0.005d
*P*-values				
Year (Y)	0.0306*	0.1111ns	0.0053**	0.0389*
Treatment (T)	< 0.0001***	0.0212*	0.0006***	0.0011**
Y × T	0.9984ns	0.4516ns	0.5337ns	0.4192ns

Values are mean ± SE. Treatment means are pooled over the two years and are based on three replicates per year. Year means are based on treatment means across the two seasons. Means within a column followed by the same letter are not significantly different (p < = 0.05) according to Duncan’s multiple range test. ns, *, **, and *** indicate non-significant and significant at p < = 0.05, p < = 0.01, and p < = 0.001, respectively

### Secondary metabolites

**Fig. 1:**
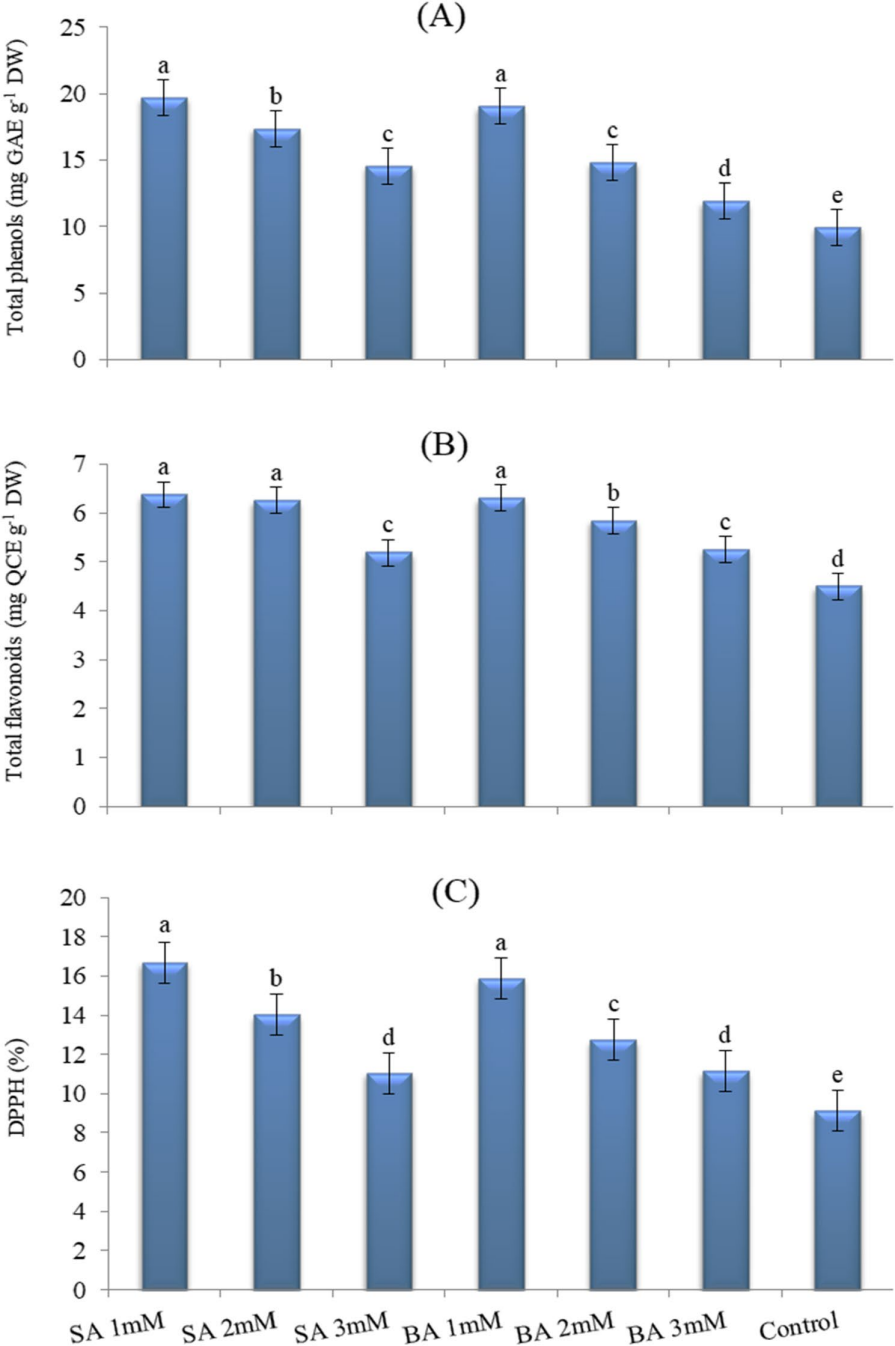
Effect of salicylic acid (SA) and benzoic acid (BA) concentrations (1, 2 and 3 mM) on the total phenols (**A**), total flavonoids (**B**) and total antioxidant activity, DPPH (**C**) of Swiss chard cv. Ruby Red. Values are means ± SE of three replicates. Means within bars followed by the same letter are not significantly different (*p* ≤ 0.05) according to Duncan’s multiple range test

Results depicted in Fig. 1 demonstrate that both SA and BA significantly increased total phenols, total flavonoids, and antioxidant activity compared to untreated plants. The Y x T interaction was significant only for total phenols, indicating that this trait showed some season-dependent treatment response. In this situation, the highest accumulation of total phenols, total flavonoids and antioxidant activity was observed with SA and BA at 1 mM, showing increments by 98.0 and 91.1%, 41.6 and 40.0% and 82.8 and 74.0%, respectively, compared to the control treatment. Similar improvement in total flavonoids was achieved by application of SA at 2 mM.

**Fig. 2:**
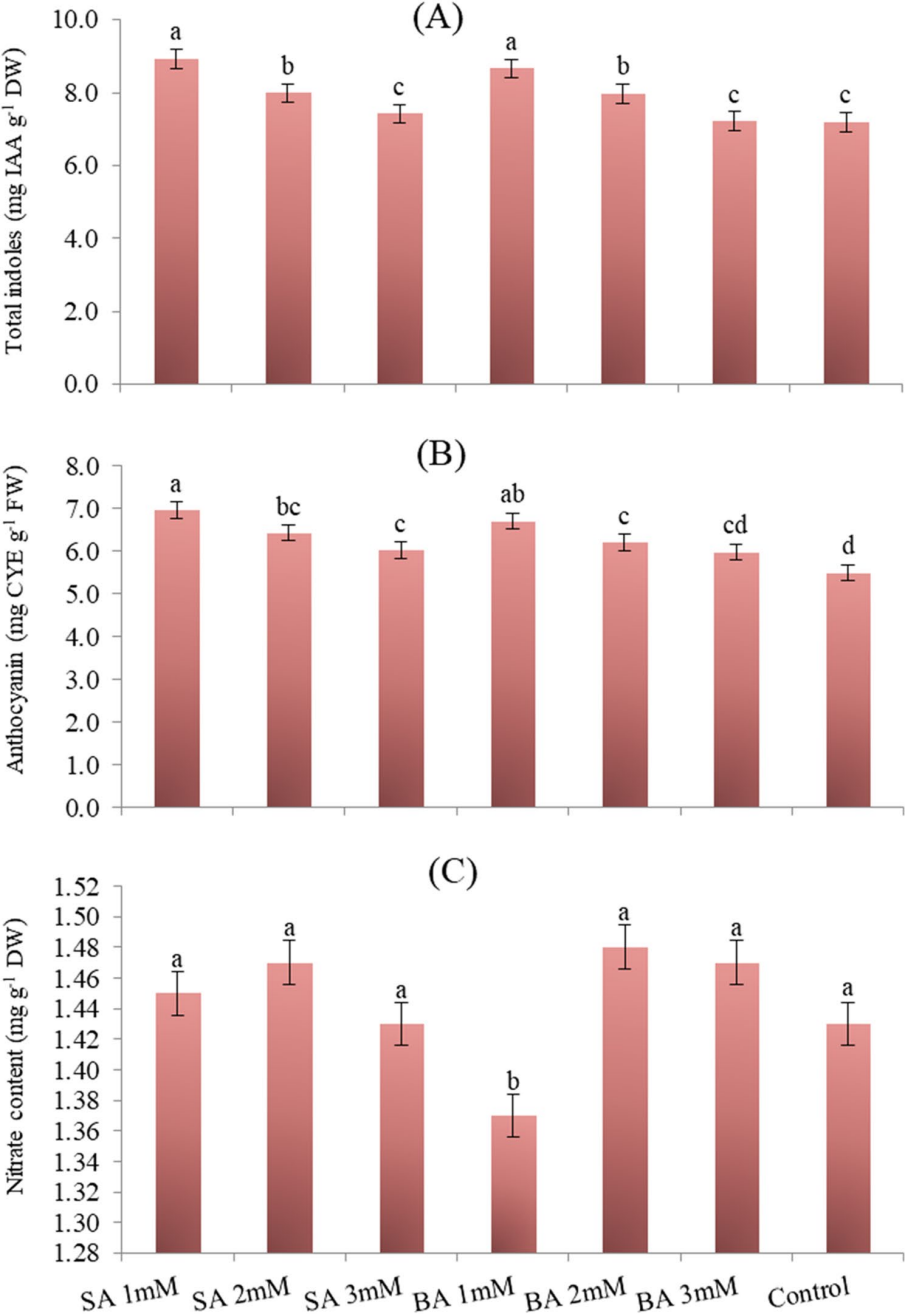
Effect of salicylic acid (SA) and benzoic acid (BA) concentrations (1, 2 and 3 mM) on the total indoles (**A**), anthocyanin (**B**) and nitrate content (**C**) of Swiss chard cv. Ruby Red. Values are means ± SE of three replicates. Means within bars followed by the same letter are not significantly different (*p* ≤ 0.05) according to Duncan’s multiple range test

As illustrated in Fig. 2, total indoles, anthocyanin and nitrate content were significantly influenced by SA and BA treatments. SA and BA at 1 mM revealed the highest significant values of total indoles and anthocyanin contents, showing increments by 24.2 and 26.8%, and 20.5 and 22.2%, respectively, compared to the control. While, BA at 1 mM was the most efficient treatment for reducing nitrate content, hence it showed the lowest nitrate concentration, which was significantly lower than SA at 2 mM and SA at 3 mM, this is desirable for leafy vegetable quality and consumer safety.

### Minerals assay

As illustrated in Table 4, nitrogen, phosphorus, and potassium contents were significantly enhanced by SA and BA treatments compared to the control. Year effect was not significant for N, P, or K, whereas treatment effects were significant for all three mineral traits. The Y x T interaction was non-significant. SA at 1 mM increased nitrogen, phosphorus, and potassium contents by 10.0, 10.5, and 9.1%, respectively, compared to the control. Moreover, BA at 1 mM revealed increments of 7.6% in nitrogen, 6.6% in phosphorus, and 9.8% in potassium, compared to the control. However, the differences between SA at 1 mM, SA at 2 mM, SA at 3 mM and BA at 1 mM for nitrogen % as well as SA at 1 mM, BA at 1 mM, BA at 2 mM and BA at 3 mM for potassium % were not significant.

**Table 4:** Effect of salicylic acid (SA) and benzoic acid (BA) concentrations (1, 2 and 3 mM) on nitrogen, phosphorus, and potassium content of Swiss chard cv. Ruby Red

Factor	Nitrogen %	Phosphorus %	Potassium %
Season			
2023/2024	2.220 ± 0.015a	0.400 ± 0.004a	3.40 ± 0.032a
2024/2025	2.200 ± 0.026a	0.390 ± 0.004a	3.34 ± 0.025a
Treatment			
SA 1mM	2.307 ± 0.012a	0.42 ± 0.005a	3.48 ± 0.025ab
SA 2mM	2.263 ± 0.017ab	0.395 ± 0.005bc	3.378 ± 0.018b
SA 3mM	2.222 ± 0.047abc	0.387 ± 0.006 cd	3.228 ± 0.041c
BA 1mM	2.258 ± 0.015ab	0.405 ± 0.003b	3.503 ± 0.027a
BA 2mM	2.188 ± 0.019bcd	0.397 ± 0.003bc	3.44 ± 0.074ab
BA 3mM	2.138 ± 0.045 cd	0.375 ± 0.003e	3.402 ± 0.035ab
Control	2.098 ± 0.041d	0.38 ± 0.003de	3.19 ± 0.02c
P-values			
Year (Y)	0.2447ns	0.2522ns	0.0981ns
Treatment (T)	0.0253*	0.0097**	0.0005***
Y × T	0.4014ns	0.2759ns	0.7026ns

Values are mean ± SE. Treatment means are pooled over the two years and are based on three replicates per year. Year means are based on treatment means across the two seasons. Means within a column followed by the same letter are not significantly different (p < = 0.05) according to Duncan’s multiple range test. ns, *, **, and *** indicate non-significant and significant at p < = 0.05, p < = 0.01, and p < = 0.001, respectively

### Pearsons’s correlation

**Fig. 3:**
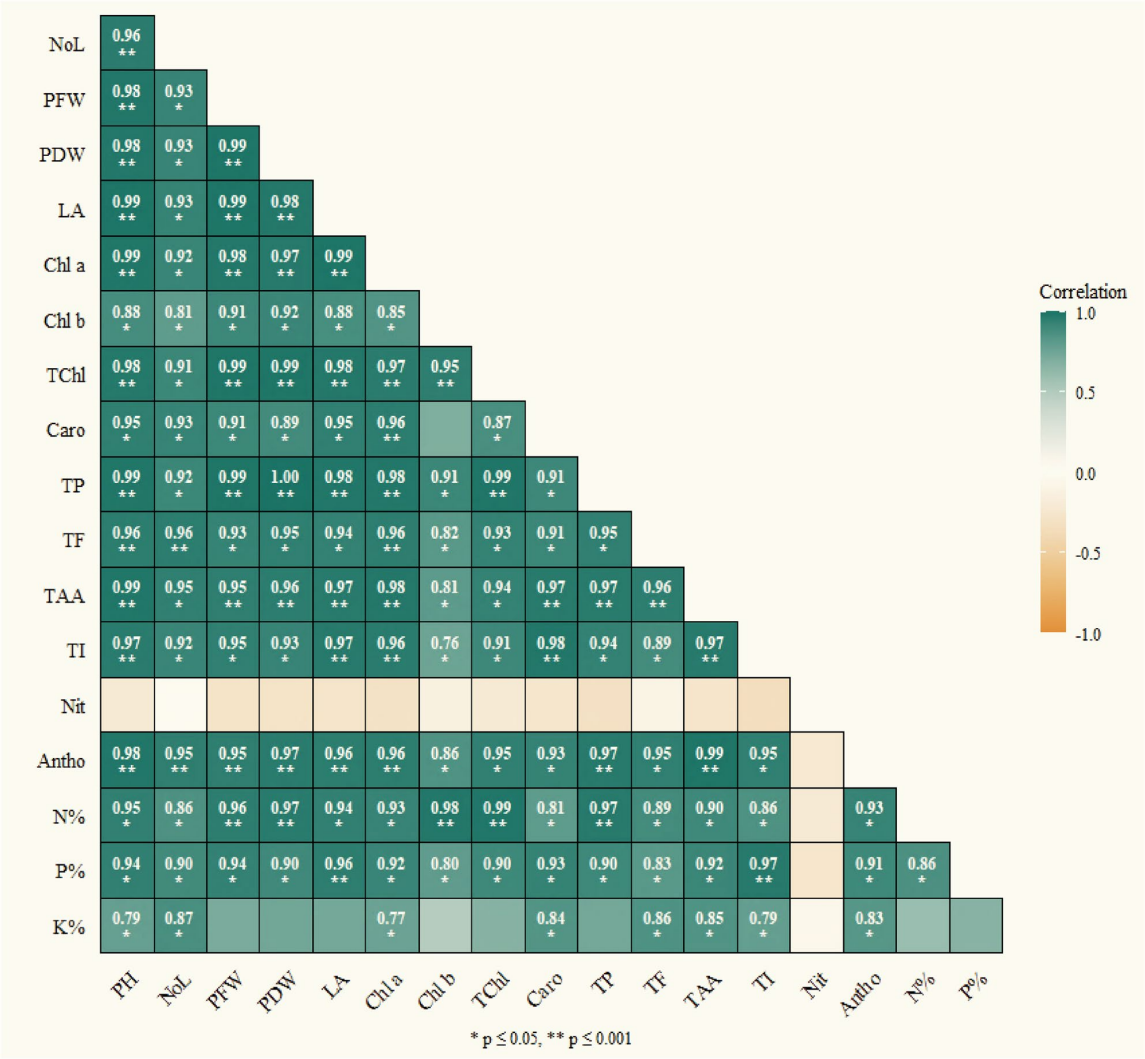
Heat map correlation coefficients between different pairs of Swiss chard characteristics treated with salicylic acid (SA) and benzoic acid (BA) foliar applications. *, ** and ***: correlation is significant at 0.05, 0.01 and 0.001 level of significance. PH (plant height), NOL (number of leaves/plant), PFW (plant fresh weight), PDW (plant dry weight), LA (leaf area), Chl a (chlorophyll a), Chl b (chlorophyll b), TChl (total chlorophyll), Caro (carotenoids), TP (total phenols), TF (total flavonoids), TAA (total antioxidant activity), TI (total indoles), Nit (nitrate content), Antho (anthocyanin), N% (nitrogen content), P% (phosphorus content), K% (potassium content)

Pearson’s correlation analysis revealed predominantly strong and positive associations among the evaluated Swiss chard traits under salicylic acid and benzoic acid foliar applications (Fig. 3). Growth-related traits were highly interrelated (*p* < 0.01), with plant height showing strong positive correlations with number of leaves (*r* = 0.96), plant fresh weight (*r* = 0.98), plant dry weight (*r* = 0.98), and leaf area (*r* = 0.99). Similarly, plant fresh weight was strongly associated with plant dry weight (*r* = 0.99) and leaf area (*r* = 0.99), indicating close coordination among vegetative growth parameters.

Photosynthetic pigment traits were also strongly correlated ((*p* < 0.01) with growth performance. Chlorophyll a was highly correlated ((*p* < 0.01) with plant height and leaf area (*r* = 0.99 for both), while total chlorophyll showed strong positive correlations with plant fresh weight, plant dry weight, and leaf area (*r* = 0.99 for each). Chlorophyll b showed comparatively lower, but still positive, associations with the other traits, particularly with total chlorophyll (*r* = 0.95), leaf area (*r* = 0.92), and plant fresh and dry weights (*r* = 0.91 for both).

The biochemical and antioxidant-related traits displayed strong positive interrelationships. Total phenols were highly correlated ((*p* < 0.01) with plant dry weight (*r* = 1.00), total chlorophyll (*r* = 0.99), plant fresh weight (*r* = 0.99), and plant height (*r* = 0.99). Total antioxidant activity was strongly associated with anthocyanin content (*r* = 0.99), total phenols (*r* = 0.97), carotenoids (*r* = 0.97), total indoles (*r* = 0.97), and chlorophyll a (*r* = 0.98). Total indoles were also strongly correlated with carotenoids (*r* = 0.98), total antioxidant activity (*r* = 0.97), and leaf area (*r* = 0.97).

Mineral composition showed positive associations with both growth and physiological traits. Nitrogen content was strongly correlated with total chlorophyll (*r* = 0.99), chlorophyll b (*r* = 0.98), plant dry weight (*r* = 0.97), total phenols (*r* = 0.97), and plant fresh weight (*r* = 0.96). Phosphorus content was strongly associated with total indoles (*r* = 0.97), leaf area (*r* = 0.96), plant height and plant fresh weight (*r* = 0.94 for both), and carotenoids (*r* = 0.93). Potassium content showed comparatively moderate positive correlations, with the highest associations observed with number of leaves (*r* = 0.87), total flavonoids (*r* = 0.86), total antioxidant activity (*r* = 0.85), carotenoids (*r* = 0.84), and anthocyanin content (*r* = 0.83). Overall, the correlation matrix indicated that improved vegetative growth was closely linked with enhanced photosynthetic pigment accumulation, antioxidant capacity, secondary metabolite content, and mineral status in Swiss chard.

On the other hand, nitrate content showed non-significant correlations with the majority of the studied parameters, indicating an inverse relationship with plant growth performance and secondary metabolite accumulation. In addition, K% showed non-significant correlations with plant fresh weight, plant dry weight, leaf area, chlorophyll b, total chlorophyll, total phenols, nitrogen % and potassium %.

### Principle component analysis (PCA)

**Fig. 4:**
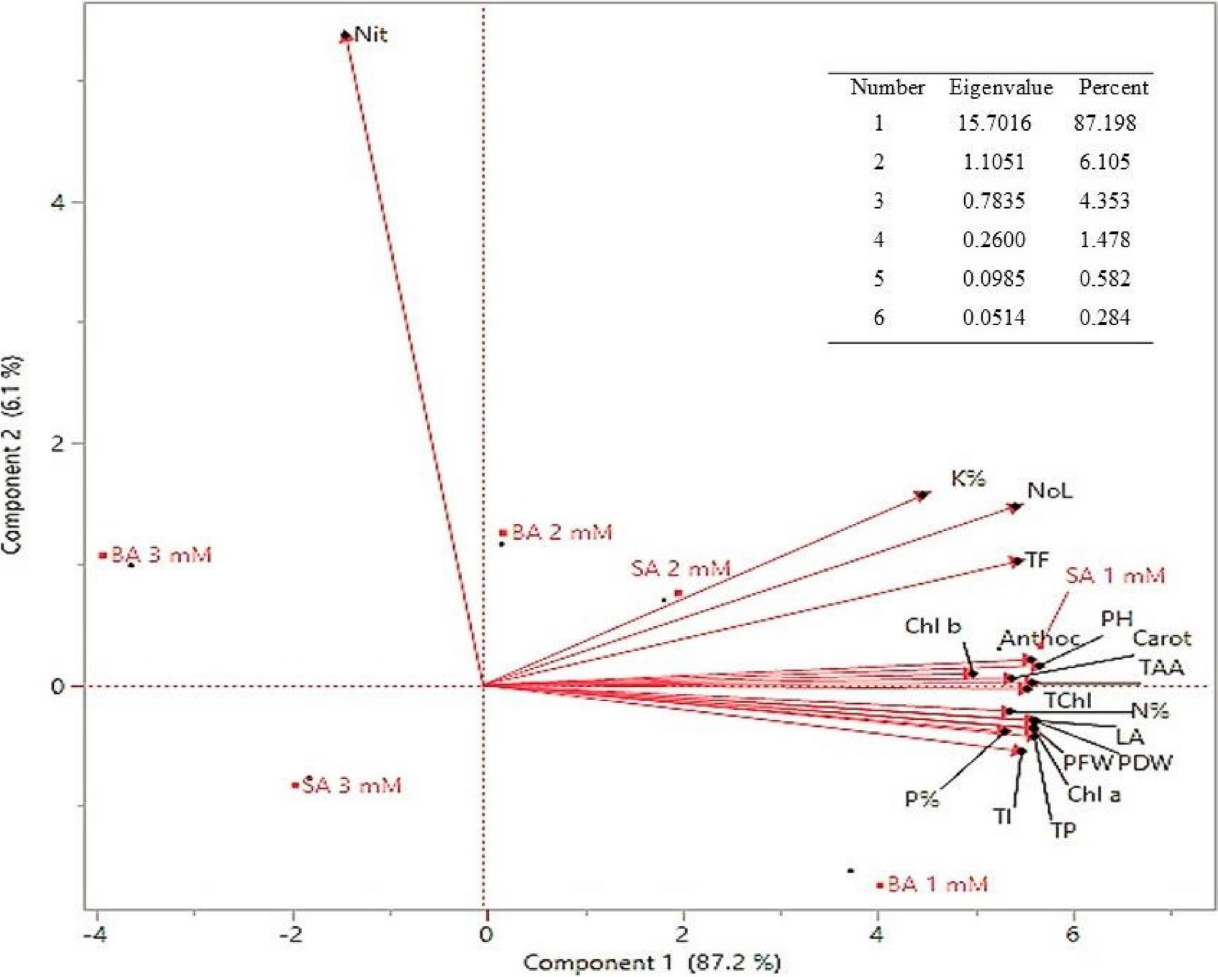
Principal component analysis (PCA) based on correlation matrix of effect of foliar applications of salicylic acid (SA) and benzoic acid (BA) on Swiss chard vegetative growth characters, mineral analysis of leaves and biochemical constituents of both seasons. Abbreviations: *PH* plant height, *NOL* number of leaves/plant, *PFW* plant fresh weight, *PDW* plant dry weight, *LA* leaf area, *Chl a* chlorophyll a, *Chl b* chlorophyll b, *TChl* total chlorophyll, *Caro* carotenoids, *TP* total phenols, *TF* total flavonoids, *TAA* total antioxidant activity, *TI* total indoles, *Nit* nitrate content, *Antho* anthocyanin, *N%* nitrogen content, *P%* phosphorus content, *K%* potassium content

Principal component analysis was conducted to assess the effect of exogenous salicylic acid and benzoic acid foliar application on the vegetative growth characteristics, chlorophyll content, secondary metabolites, in terms of, total phenols and total flavonoids, total indoles, total antioxidant activity, anthocyanin content, and mineral analysis of leaves. PCA was performed on centered and scaled variables using the correlation matrix because the measured traits had different units. PCA results based on the correlation matrix are presented in (Fig. 4). Each variable is illustrated by an arrow, and the longer its length, the greater its contribution to a given component. The angle formed by the arrows indicates the degree of correlation between variables; the smaller the angle, the greater the correlation. It was found that the first two principal components accounted for 87.2 and 6.1% of the variations for Component 1 and Component 2, respectively. The cumulative proportion of the variation approached 93.3% of the total variance. On the other hand, the remaining components jointly explained 6.7% of the variance. Based on 18 standardized variables, the approximate eigenvalues of PC1 and PC2 were 15.70 and 1.10, respectively. Most of the examined traits were discriminated by Component 1, and thus explained by the larger proportion of variance (87.2%). The high PC1 contribution is attributable to the strong positive covariance among most growth, pigment, secondary metabolite, and mineral variables, as shown by the correlation heat map.

It is clearly noted that all evaluated traits, except nitrate, were positively associated with SA and BA at 1 and 2 mM treatments. The SA and BA at 3 mM treatments were positioned on the negative range, which means that it gave the lowest values of all tested parameters compared with the foliar applications of SA and BA.

## Discussion

The superiority of 1 mM SA, or BA for the vegetative growth parameters enhancement may be attributed to the optimal activation of physiological processes such as cell division, elongation, and assimilates translocation. Moreover, it has been reported that SA interacts with other hormones that regulate cell division and expansion, including auxin, gibberellins, and ethylene to modulate plant growth [39, 40]. SA affects the root development through modulating auxin accumulation and transport. Additionally, it is clear from results that the foliar applications of SA or BA at 1mM significantly increased the total indoles that may enhance the root development which is reflected on the vegetative growth parameters. Further, SA is a signaling molecule that modulates stomatal conductance, nutrient uptake, and enzymatic activities linked to primary metabolism, thereby enhancing plant growth under both normal and stress conditions [14, 15, 41]. In addition, SA application modulates the nitric oxide content in plant cells that regulates the plant defense responses and growth metabolism [42–44]. Furthermore, BA is a precursor in SA biosynthesis, enabling analogous physiological responses as SA.

On the other hand, the decline of plant growth at the high concentrations of SA and BA applications may be a result of the high accumulation of SA or BA in the plant cells that induces metabolic imbalance or oxidative stress, which can disturb the cell homeostasis and inhibit growth [45].

The increments in chlorophyll a, chlorophyll b, and total chlorophyll with 1 mM treatments of SA or BA indicate improved photosynthetic efficiency. This can be ascribed to the function of SA in stabilizing chloroplast structure, enhancing Rubisco activity [46], and reducing chlorophyll degradation [47]. Moreover, the enhancement in carotenoids content represents an improvement in photoprotection [48, 49]; hence, these pigments play a role in scavenging the reactive oxygen species (ROS) which could harm plant cell membrane, especially at high accumulation [50–52]. While the decrements showed at higher concentrations indicate that the excess of phenolic regulators may hinder chlorophyll biosynthesis or accelerate pigment degradation [53].

Concerning the secondary metabolites accumulation and antioxidant activity in response to SA and BA, the increment in total phenols, flavonoids, and antioxidant activity, particularly with 1 mM treatments, may be a result of activation of the phenylpropanoid pathway. Since SA regulates key enzymes, i.e., phenylalanine ammonia-lyase, leading to an enhancement in the biosynthesis of phenolic compounds [13, 14, 18]. These metabolites serve as efficient antioxidants, which have roles in scavenging ROS and protecting cellular components from oxidative damage [54–57]. Moreover, SA serves as a vital regulator of plant redox homeostasis, leading to an increase in the ratio of reduced glutathione to oxidized glutathione, thereby improving the antioxidant capacity of the cell and enhancing the activity of antioxidant enzymes in the ascorbate-glutathione cycle under both stress and non-stress conditions [58, 59]. The glutathione functions as a sulfur donor and a cofactor for enzymes engaged in stress responses and the production of protective compounds. It acts at the intersection of primary and secondary metabolism, where its levels often influence the accumulation of metabolites, i.e., glucosinolates, flavonoids, and phenylpropanoids [60].

The effects of SA and BA on the enhancement of bioactive compounds, i.e., total indoles and anthocyanin, followed the same trend as that of the secondary metabolites. The increase in indole compounds may be associated with stimulation of tryptophan-dependent pathways, while elevated anthocyanin content reflects activation of flavonoid biosynthesis [61, 62].

Reducing nitrate content is a critical aim for maintaining high quality of leafy crops [63]. Herein, the enhancement of N, P, and K % associated with SA 1mM may be attributed to the role of SA in enhancing nitrogen assimilation through the activation of key enzymes such as nitrate reductase and glutamine synthetase [20, 64]. Further, SA improves the root growth and membrane permeability, thereby facilitating greater nutrient uptake from the soil [65], the obtained results emphasize the role of SA on the total indoles content which is reflected on the root growth and development [66].

Although the responses were primarily attributed to SA/BA signaling and phenylpropanoid-related regulation, solvent and surfactant effects must be considered. This potential bias was minimized by using the same final DMSO and Tween 20 concentrations in the control and treated solutions. Therefore, the observed treatment differences are more likely attributable to SA and BA concentrations rather than to differential solvent or Tween 20 that affects cuticular penetration.

## Conclusions

The findings of the current work have revealed new insights of the potential application of low-concentration of benzoic acid to enhance the production of secondary metabolites, thereby contributing to the growth of Swiss chard. Overall, the findings indicate that foliar application of salicylic acid and benzoic acid, particularly at 1 mM, promotes Swiss chard productivity through coordinated physiological and biochemical mechanisms. In this case, the application of 1 mM benzoic acid was comparable to that of the same concentration of salicylic acid in achieving maximum leafy yield, as evidenced by increased leaf weight and number per plant, while showing a distinct advantage in reducing nitrate accumulation. These responses are likely mediated by enhanced photosynthetic pigment accumulation, improved mineral uptake, acquisition and utilization, strengthened antioxidant capacity, and stimulation of secondary metabolite biosynthesis through activation of phenylpropanoid/flavonoid pathways, indole-related growth regulation, resulting in greater biomass production and nutritional quality, whereas higher concentrations may disturb metabolic homeostasis and reduce growth performance. For producers, BA at 1 mM represents a simple and potentially cost-effective foliar strategy to improve marketable yield and nutritional quality of Swiss chard with low nitrate accumulation, which is desirable for leafy vegetable quality and consumer safety. However, further specialized molecular studies are needed to elucidate the relationship between benzoic acid concentration and gene expression to understand its potential positive/negative effects on plant growth. 

## Supplementary Information

Supplementary Material 1.

## Data Availability

No datasets were generated or analysed during the current study.
