# Supplementary material for: Benzoic acid versus salicylic acid efficacy in regulating growth, accumulation of secondary metabolites and NO3 content of Swiss chard (Beta vulgaris L. var. cicla)
Source: BMC Plant Biol. 2026 Jul 28;26:1277. doi: 10.1186/s12870-026-09593-5 (PMC13418131; doi:10.1186/s12870-026-09593-5)
Supplement: Supplementary file 1 — Supplementary Material 1. [file 12870_2026_9593_MOESM1_ESM.docx]

**Supplementary material**

**Supplementary Table S1. Combined ANOVA for year, replicate within year, treatment, and year x treatment effects on Swiss chard traits.**

| **Trait** | **Source of variation** | **df** | **MS** | **F-value** | **P-value** | **Sig.** |
| --- | --- | --- | --- | --- | --- | --- |
| Plant height | Year | 1 | 1.79 | 0.92 | 0.3922 | ns |
|  | Rep(Year) | 4 | 1.95 | 0.75 | 0.5681 | ns |
|  | Treatment | 6 | 29.85 | 11.53 | 0.0045 | ** |
|  | Year × Treatment | 6 | 2.59 | 1 | 0.4502 | ns |
|  | Error | 24 | 2.6 |  |  |  |
| Leaves number | Year | 1 | 4.44 | 10.88 | 0.03 | * |
|  | Rep(Year) | 4 | 0.41 | 1.5 | 0.2334 | ns |
|  | Treatment | 6 | 2.78 | 7.97 | 0.0116 | * |
|  | Year × Treatment | 6 | 0.35 | 1.28 | 0.3031 | ns |
|  | Error | 24 | 0.27 |  |  |  |
| Leaves fresh weight | Year | 1 | 1141.93 | 8.76 | 0.0416 | * |
|  | Rep(Year) | 4 | 130.36 | 0.24 | 0.9114 | ns |
|  | Treatment | 6 | 20027.97 | 52.26 | 0.0001 | *** |
|  | Year × Treatment | 6 | 383.21 | 0.71 | 0.6428 | ns |
|  | Error | 24 | 537.63 |  |  |  |
| Leaves dry weight | Year | 1 | 0.74 | 0.05 | 0.8404 | ns |
|  | Rep(Year) | 4 | 16.05 | 0.69 | 0.6081 | ns |
|  | Treatment | 6 | 434.75 | 23.02 | 0.0007 | *** |
|  | Year × Treatment | 6 | 18.88 | 0.81 | 0.5739 | ns |
|  | Error | 24 | 23.37 |  |  |  |
| Leaf area | Year | 1 | 9406.01 | 9.4 | 0.0374 | * |
|  | Rep(Year) | 4 | 1000.17 | 0.23 | 0.9182 | ns |
|  | Treatment | 6 | 77963.01 | 25.67 | 0.0005 | *** |
|  | Year × Treatment | 6 | 3036.91 | 0.7 | 0.6511 | ns |
|  | Error | 24 | 4328.18 |  |  |  |
| Chlorophyll a | Year | 1 | 0.01 | 10.73 | 0.0306 | * |
|  | Rep(Year) | 4 | 0.00094 | 0.64 | 0.642 | ns |
|  | Treatment | 6 | 0.04 | 364.37 | <0.0001 | *** |
|  | Year × Treatment | 6 | 0.00010 | 0.07 | 0.9984 | ns |
|  | Error | 24 | 0.00150 |  |  |  |
| Chlorophyll b | Year | 1 | 0.01 | 4.16 | 0.1111 | ns |
|  | Rep(Year) | 4 | 0.00170 | 0.35 | 0.8412 | ns |
|  | Treatment | 6 | 0.03 | 6.24 | 0.0212 | * |
|  | Year × Treatment | 6 | 0.0050 | 0.99 | 0.4516 | ns |
|  | Error | 24 | 0.0050 |  |  |  |
| Total chlorophyll | Year | 1 | 0.03 | 30.21 | 0.0053 | ** |
|  | Rep(Year) | 4 | 0.00096 | 0.16 | 0.956 | ns |
|  | Treatment | 6 | 0.13 | 24.55 | 0.0006 | *** |
|  | Year × Treatment | 6 | 0.01 | 0.87 | 0.5337 | ns |
|  | Error | 24 | 0.01 |  |  |  |
| Carotenoids | Year | 1 | 0.00320 | 9.17 | 0.0389 | * |
|  | Rep(Year) | 4 | 0.00035 | 1.02 | 0.4162 | ns |
|  | Treatment | 6 | 0.01 | 19.34 | 0.0011 | ** |
|  | Year × Treatment | 6 | 0.00036 | 1.05 | 0.4192 | ns |
|  | Error | 24 | 0.00034 |  |  |  |
| Total phenols | Year | 1 | 1.17 | 2.31 | 0.203 | ns |
|  | Rep(Year) | 4 | 0.51 | 1.58 | 0.2128 | ns |
|  | Treatment | 6 | 78.39 | 36.12 | 0.0002 | *** |
|  | Year × Treatment | 6 | 2.17 | 6.76 | 0.0003 | *** |
|  | Error | 24 | 0.32 |  |  |  |
| Total flavonoids | Year | 1 | 0.55 | 22.36 | 0.0091 | ** |
|  | Rep(Year) | 4 | 0.02 | 0.35 | 0.8404 | ns |
|  | Treatment | 6 | 3.03 | 104.86 | <0.0001 | *** |
|  | Year × Treatment | 6 | 0.03 | 0.42 | 0.8613 | ns |
|  | Error | 24 | 0.07 |  |  |  |
| DPPH antioxidant activity | Year | 1 | 4.38 | 6.56 | 0.0626 | ns |
|  | Rep(Year) | 4 | 0.67 | 1.49 | 0.2369 | ns |
|  | Treatment | 6 | 45.39 | 49.92 | 0.0001 | *** |
|  | Year × Treatment | 6 | 0.91 | 2.02 | 0.1017 | ns |
|  | Error | 24 | 0.45 |  |  |  |
| Total indoles | Year | 1 | 0.19 | 1.9 | 0.2401 | ns |
|  | Rep(Year) | 4 | 0.1 | 2.14 | 0.1063 | ns |
|  | Treatment | 6 | 2.86 | 24.82 | 0.0005 | *** |
|  | Year × Treatment | 6 | 0.12 | 2.43 | 0.0564 | ns |
|  | Error | 24 | 0.05 |  |  |  |
| Nitrate | Year | 1 | 0.000086 | 0.3 | 0.613 | ns |
|  | Rep(Year) | 4 | 0.00029 | 0.08 | 0.9878 | ns |
|  | Treatment | 6 | 0.01 | 6.02 | 0.0231 | * |
|  | Year × Treatment | 6 | 0.00140 | 0.38 | 0.8836 | ns |
|  | Error | 24 | 0.00360 |  |  |  |
| Anthocyanin | Year | 1 | 2.07 | 12.61 | 0.0238 | * |
|  | Rep(Year) | 4 | 0.16 | 1.45 | 0.2492 | ns |
|  | Treatment | 6 | 1.45 | 35.28 | 0.0002 | *** |
|  | Year × Treatment | 6 | 0.04 | 0.36 | 0.8951 | ns |
|  | Error | 24 | 0.11 |  |  |  |
| Nitrogen | Year | 1 | 0.01 | 1.86 | 0.2447 | ns |
|  | Rep(Year) | 4 | 0.00280 | 0.54 | 0.7062 | ns |
|  | Treatment | 6 | 0.03 | 5.79 | 0.0253 | * |
|  | Year × Treatment | 6 | 0.01 | 1.08 | 0.4014 | ns |
|  | Error | 24 | 0.01 |  |  |  |
| Phosphorus | Year | 1 | 0.00086 | 1.79 | 0.2522 | ns |
|  | Rep(Year) | 4 | 0.00048 | 3.92 | 0.0137 | * |
|  | Treatment | 6 | 0.00140 | 8.58 | 0.0097 | ** |
|  | Year × Treatment | 6 | 0.00017 | 1.35 | 0.2759 | ns |
|  | Error | 24 | 0.00012 |  |  |  |
| Potassium | Year | 1 | 0.04 | 4.62 | 0.0981 | ns |
|  | Rep(Year) | 4 | 0.01 | 1.53 | 0.2261 | ns |
|  | Treatment | 6 | 0.09 | 26.39 | 0.0005 | *** |
|  | Year × Treatment | 6 | 0.00330 | 0.63 | 0.7026 | ns |
|  | Error | 24 | 0.01 |  |  |  |

*Notes: Y = year; T = treatment. Year was tested against Rep(Year), treatment against Y x T, and Y x T against residual error. Sig.: ns = non-significant; *, **, and *** denote significance at p <= 0.05, p <= 0.01, and p <= 0.001, respectively.*

| **Trait** | **PH** | **NoL** | **PFW** | **PDW** | **LA** | **Chl a** | **Chl b** | **TChl** | **Caro** | **TP** | **TF** | **TAA** | **TI** | **Nit** | **Antho** | **N%** | **P%** |
| --- | --- | --- | --- | --- | --- | --- | --- | --- | --- | --- | --- | --- | --- | --- | --- | --- | --- |
| **NoL** | 0.96** |  |  |  |  |  |  |  |  |  |  |  |  |  |  |  |  |
| **PFW** | 0.98** | 0.93* |  |  |  |  |  |  |  |  |  |  |  |  |  |  |  |
| **PDW** | 0.98** | 0.93* | 0.99** |  |  |  |  |  |  |  |  |  |  |  |  |  |  |
| **LA** | 0.99** | 0.93* | 0.99** | 0.98** |  |  |  |  |  |  |  |  |  |  |  |  |  |
| **Chl a** | 0.99** | 0.92* | 0.98** | 0.97** | 0.99** |  |  |  |  |  |  |  |  |  |  |  |  |
| **Chl b** | 0.88* | 0.81* | 0.91* | 0.92* | 0.88* | 0.85* |  |  |  |  |  |  |  |  |  |  |  |
| **TChl** | 0.98** | 0.91* | 0.99** | 0.99** | 0.98** | 0.97** | 0.95** |  |  |  |  |  |  |  |  |  |  |
| **Caro** | 0.95* | 0.93* | 0.91* | 0.89* | 0.95* | 0.96** | ns | 0.87* |  |  |  |  |  |  |  |  |  |
| **TP** | 0.99** | 0.92* | 0.99** | 1.00** | 0.98** | 0.98** | 0.91* | 0.99** | 0.91* |  |  |  |  |  |  |  |  |
| **TF** | 0.96** | 0.96** | 0.93* | 0.95* | 0.94* | 0.96** | 0.82* | 0.93* | 0.91* | 0.95* |  |  |  |  |  |  |  |
| **TAA** | 0.99** | 0.95* | 0.95** | 0.96** | 0.97** | 0.98** | 0.81* | 0.94* | 0.97** | 0.97** | 0.96** |  |  |  |  |  |  |
| **TI** | 0.97** | 0.92* | 0.95** | 0.93* | 0.97** | 0.96** | 0.76* | 0.91* | 0.98** | 0.94* | 0.89* | 0.97** |  |  |  |  |  |
| **Nit** | ns | ns | ns | ns | ns | ns | ns | ns | ns | ns | ns | ns | ns |  |  |  |  |
| **Antho** | 0.98** | 0.95** | 0.95** | 0.97** | 0.96** | 0.96** | 0.86* | 0.95* | 0.93* | 0.97** | 0.95* | 0.99** | 0.95* | ns |  |  |  |
| **N%** | 0.95* | 0.86* | 0.96** | 0.97** | 0.94* | 0.93* | 0.98** | 0.99** | 0.81* | 0.97** | 0.89* | 0.90* | 0.86* | ns | 0.93* |  |  |
| **P%** | 0.94* | 0.90* | 0.94* | 0.90* | 0.96** | 0.92* | 0.80* | 0.90* | 0.93* | 0.90* | 0.83* | 0.92* | 0.97** | ns | 0.91* | 0.86* |  |
| **K%** | 0.79* | 0.87* | ns | ns | ns | 0.77* | ns | ns | 0.84* | ns | 0.86* | 0.85* | 0.79* | ns | 0.83* | ns | ns |

**Supplementary Table S2. Pearsons’s correlation table,**
